# Comparative Health Assessments of Alaskan Ice Seals

**DOI:** 10.3389/fvets.2019.00004

**Published:** 2019-02-06

**Authors:** Caroline E. C. Goertz, Colleen Reichmuth, Nicole M. Thometz, Heather Ziel, Peter Boveng

**Affiliations:** ^1^Alaska SeaLife Center, Seward, AK, United States; ^2^Institute of Marine Sciences, University of California, Santa Cruz, Santa Cruz, CA, United States; ^3^Department of Biology, University of San Francisco, San Francisco, CA, United States; ^4^Polar Ecosystems Program, Marine Mammal Laboratory, Alaska Fisheries Science Center, National Oceanic and Atmospheric Administration, Seattle, WA, United States

**Keywords:** *Phoca largha*, *Histriophoca fasciata*, *Erignathus barbatus*, *Pusa hispida*, hematology, serology, fecal pathogen, parasite

## Abstract

Bearded (*Erignathus barbatus*), ringed (*Pusa hispida*), spotted (*Phoca largha*), and ribbon (*Histriophoca fasciata*) seals rely on seasonal sea-ice in Arctic and sub-Arctic regions. Many aspects of the biology and physiology of these seals are poorly known, and species-typical health parameters are not available for all species. Such information has proven difficult to obtain due to the challenges of studying Arctic seals in the wild and their minimal historic representation in aquaria. Here, we combine diagnostic information gathered between 2000 and 2017 from free-ranging seals, seals in short-term rehabilitation, and seals living in long-term human care to evaluate and compare key health parameters. For individuals in apparent good health, hematology, and blood chemistry values are reported by the source group for 10 bearded, 13 ringed, 73 spotted, and 81 ribbon seals from Alaskan waters. For a smaller set of individuals handled during veterinary or necropsy procedures, the presence of parasites and pathogens is described, as well as exposure to a variety of infectious diseases known to affect marine mammals and/or humans, with positive titers observed for *Brucella, Leptospira*, avian influenza, herpesvirus PhHV-1, and morbillivirus. These data provide initial baseline parameters for hematology, serum chemistries, and other species-level indicators of health that can be used to assess the condition of individual seals, inform monitoring and management efforts, and guide directed research efforts for Alaskan populations of ice-associated seals.

## Introduction

Rapid environmental change threatens the stability and overall health of Arctic and sub-Arctic ecosystems (1–3). For long-lived and highly-derived species such as ice-associated marine mammals, the unprecedented rate of sea ice loss in northern latitudes may be especially devastating (4–6). A recent unusual mortality event (UME) in Alaska highlighted the potential sensitivity of Arctic and sub-Arctic pinnipeds to the effects of climate change. The Alaska Northern Pinniped UME (2011–2018) was defined by seals and walruses presenting with abnormal behavior, suspected disrupted molts, and skin lesions (7); however, the primary cause of mortality remains unknown (8, 9). The UME syndrome extended across a circumpolar arc, with cases from the Beaufort Sea, Chukchi Sea, Bering Strait, and Bering Sea in Alaska, as well as in other regions of the Arctic Ocean extending to the North Atlantic Ocean (8). The transboundary nature of this UME increases concern that the opening of northwest and northeast passages through the Arctic, created by retreating sea ice, may result in disease transmission and possible epidemics in Alaskan marine mammals [see (10)]. This concern appears to be well founded, as nucleic acids from, and antibodies to, phocine distemper virus (PDV)—a pathogen responsible for two epidemics in northern Europe—have been detected for the first time in the North Pacific Ocean within the last decade (11), coincident with the first seasonal ice-free access routes through the Arctic from the North Atlantic Ocean (12).

In Alaska, there are four species of ice-associated seals: spotted (*Phoca largha*), ribbon (*Histriophoca fasciata*), bearded (*Erignathus barbatus*), and ringed (*Pusa hispida*) seals, often referred to collectively as “ice seals.” All four species utilize sea ice during key life-history stages; however, they vary greatly in their use of, and dependence on, seasonal sea ice throughout the year. Spotted and ribbon seals use the marginal edge of the pack ice as a platform for pupping and molting annually, but individuals of these species are not generally associated with sea ice year-round (13, 14). Spotted seals use terrestrial haul-outs between foraging bouts during the summer and fall (15), while ribbon seals, the presumed deepest diving of the four species, maintain a predominately pelagic lifestyle aside from pupping and molting periods (14, 16, 17). In contrast, bearded and ringed seals are more frequently associated with sea ice throughout the year (18, 19). Bearded seals use broken and moving pack ice as a platform for pupping and molting each spring (20, 21) and are typically found on ice floes over shallow areas due to their benthic foraging habits (22, 23). Throughout much of their breeding range, ringed seals use areas of shore-fast ice, where they maintain breathing holes, and excavate subnivian lairs for pupping, nursing, and to avoid predation by polar bears. Ringed seals remain in areas of extensive fast ice for much of the year and haul out on exposed sea ice for extended periods only during the spring molt (21, 24, 25). Given the distinct ways in which Alaskan ice seals use and rely on seasonal sea ice, rapidly changing conditions in Arctic and sub-Arctic ecosystems are likely to influence these species in different ways. Such changes could relate to geographic distribution, nutritional status, pathogen exposure, and other risk factors.

There are few baseline studies available for Alaskan ice seals that could provide the critical reference values needed to assess the health of individuals and populations [for review see (10, 26)]. This is particularly true with respect to normal hematology and clinical chemistries. There are presently no published blood panels available for spotted or bearded seals. Lenfant et al. (27) reports some hematology parameters pertaining to blood oxygen storage for five ribbon seals, but no other data are available for this poorly studied species. Relatively more information is available for ringed seals. There are some data available concerning the number and type of cells present in the blood of both captive (28) and free-ranging ringed seals (29, 30), and several plasma chemistry parameters from a subset of the same wild individuals (31). A few plasma chemistry values are also reported for several captive ringed seals (28, 31). However, there is no complete data set concerning baseline blood hematology and chemistry parameters for any Alaskan ice seal.

Documentation of disease exposure and parasites in Alaskan ice seals has come primarily from healthy seals taken during subsistence hunting or handled during field research activities. These data have been reported and recently summarized in comprehensive species reviews and research reports for spotted (13, 32), ribbon (14, 33), bearded (19, 34), and ringed seals (18, 35). Microbial isolates from the target species are not commonly reported. In contrast, blood, disease, and other health parameters are readily available for more temperate-living harbor seals (*Phoca vitulina*) (36–41) whose northern range in the Pacific overlaps with that of spotted seals in the southern portion of their range.

To better understand the species-typical health and physiology of Alaskan ice seals, we describe hematological parameters, blood chemistries, serology, microbial isolates, and parasite exposure data for all four species. These data, obtained from free-ranging seals sampled during field assessments, stranded seals in short-term rehabilitation and/or at necropsy, and healthy seals living in long-term human care, provide initial reference parameters for assessing the health and overall condition of both wild and captive ice seals.

## Methods

### Field-Based Studies

#### Source of Animals and Handling

In the spring of 2007–2010, 2014, and 2016, 80 ribbon seals and 62 spotted seals were captured at the edge of the pack ice of the Bering Sea using long-handled salmon landing nets and handled on the ice floes on which they were caught. Seals were physically restrained if <1 y old, or lightly sedated with diazepam or midazolam (0.1 mg/kg) injected intravenously into the extradural intravertebral vein. Prior to release, seals were tagged, samples were collected, and the effects of sedation were reversed with an intramuscular injection of flumazenil (0.01 mg/kg) if needed. In June 2009 and 2011, and July 2012, eight bearded seals were captured in pack ice in Kotzebue Sound, Alaska using tangle nets deployed from boats. The seals were moved to adjacent ice floes for short-term handling using the same methods as described for ribbon and spotted seals. Morphological characteristics, including size and coat condition, were used to distinguish between age groups.

#### Sample Collection, Processing, and Storage

Blood was collected from the extradural intravertebral vein (29) using 18-gauge, 1.5–3.5 inch needles, depending on the size of the seal, into SST and K_2_EDTA-coated Vacutainer® tubes (Becton Dickinson, Franklin Lakes, New Jersey). Blood tubes were kept in insulated containers to prevent freezing before processing. Blood was processed within 6 h of collection on the main research ship (spotted and ribbon seals) or at a shore-based laboratory (bearded seals). Serum separator tubes were centrifuged for 10 min at 10,000 rpm, and 1–2 ml aliquots of serum were transferred to vials and stored at −80°C until analysis.

#### Laboratory Analysis

Blood samples from 62 spotted seals, 80 ribbon seals, and 8 bearded seals (Table 1) were analyzed for a subset of hematological parameters. Hematocrit (Hct) was determined in duplicate for each seal by filling two heparinized microhematocrit capillary tubes with whole blood from each sample and centrifuging for 10 min at 10,000 rpm. Samples were read with a microhematocrit capillary tube reader card (±1%). Hemoglobin concentration (Hb) was measured in duplicate using the cyanmethemoglobin method (Pointe Scientific, Inc.). A 10 μl aliquot of whole blood was added to 2.0 ml of cyanmethemoglobin reagent and stored in cryovials covered in aluminum foil at room temperature. Hemoglobin concentration was measured at the end of the field season (within 2 months of collection) with a Thermo Spectronic BioMate 3 spectrophotometer at λ of 540 nm. Mean corpuscular hemoglobin concentration (MCHC; average concentration of hemoglobin in a given amount of packed red blood cells) was calculated as (Hb/Hct)^*^100.

**Table 1:** Summary hematology data for live-sampled spotted seals, ribbon seals, bearded seals, and ringed seals.

**Source population**	**Spotted seal**			**Ribbon seal**		**Bearded seal**		**Ringed seal**	
	**Rehabilitated: ASLC**	**Captive: LML**	**Wild caught: bering sea (MML)**	**Rehabilitated: ASLC**	**Wild caught: bering sea (MML)**	**Wild caught: ASLC**	**Wild caught: chukchi sea, bering sea (MML)**	**Rehabilitated: ASLC**	**Captive: ASLC, LML**
Hematology values	Total range	Total range	5–95% range	Total range	5–95% range	Total range	Total range	Total range	Total range
Number individuals	*n* = 9	*n* = 2	*n* = 62	*n* = 1	*n* = 80	*n* = 2	*n* = 5–8	*n* = 10	*n* = 3
Number samples	2–18	4–6	62	2–3	79–80	4	5–8	8–19	6
Individuals—age	<1.5 y	3–5 y	Mixed^a^	1 adult	Mixed^b^	<1 y	mixed^c^	<1.5 y	mixed^d^
Individuals—sex	4 F, 5 M	2 M	33 F, 29 M	1 F	39 F, 41 M	2 M	4 F, 4 M	3 F, 7 M	1 F, 2 M
RBC (10^6^/mm^3^)	3.6–5.3	4.6–5.4		4.3–5.1		3.6–5.0		4.1–5.2	3.9–6.0
Hb (g/dl)	13–23	17–24	13–24^e^	29–34	13–33^e^	23–28	19–32^e^	15–26	14–30
HCT ^f^ (%)	32–55	52–64	45–62	68–74	45–76	57–65	52–57	39–60	42–68
MCV (fl)	79–113	101–121		140–141		119–140		98–133	95–130
MCH (pg)	30–51	37–45		66–67		55–65		38–52	33–50
MCHC (g/dl)	35–46	32–41	30–42	47–48	22–48	45–47	35–58	39–44	34–45
Platelets^g^(10^3^/mm^3^)	111–869	261–572		35–124		243–402		36–467	74–412
Leukocytes /μl	4,300–10,800	5,100–8,420		5,700–8,500		9,150–12,300		7,400–16,800	6,400–13,000
Neutrophil (band) /μl	0–158	0–0		0–0		0–0		0–354	0–0
Neutrophil (mature) /μl	1,806–7,560	1,224–4,884		4,218–6,035		5,582–9,401		2,966–8,409	2,405–4,158
Lymphocyte^h^ /μl	972–3,456	1,616–3,021		513–1,079		1,007–1,845		950–4,233	2,368–5,670
Monocyte /μl	120–1,349	318–1,095		798–1,328		369–2,507		366–2,185	195–1,149
Eosinophil /μl	0–472	0–848		228–747		119–1,107		0–4,896	120–2,142
Basophil/ μl	0–864	0–337		0–0		0–246		0–2,352	−4

*For each source population, the reported values (total range for data sets with n < 30 or 5–95% range for data sets with n > 30), numbers of individuals sampled (n), number of samples evaluated, ages of sampled individuals, and sexes of sampled individuals are provided. Source populations of sampled seals are from rehabilitation (Alaska SeaLife Center, ASLC), long-term captive (ASLC or Long Marine Lab, LML), or wild capture (ASLC or NOAA Marine Mammal Lab, MML). Hematology parameters were measured either manually or on one of several analyzers (see notations in text); thus, these can be considered combined, descriptive data, or viewed quantitatively by subgroup. Data for individual animals within each subject group are provided in Supplementary Table 1*.

a *Age distribution of spotted seals wild-caught in the Bering Sea: 13 adults, 13 subadults, 29 young-of-year, 7 pups*.

b *Age distribution of ribbon seals wild-caught in the Bering Sea: 33 adults, 22 subadults, 19 young-of-year, 6 pups*.

c *Age distribution of bearded seals wild-caught in the Chukchi Sea, Bering Sea: 1 adult, 6 subadults, 1 pup*.

d *Age distribution of ringed seals sampled in long-term captivity, Alaska SeaLife Center, and Long Marine Lab: 1 subadult, 2 adults*.

e *Hemoglobin measured using cyanmethemoglobin method*.

f *HCT is reported as the directly measured packed cell volume (PCV)*.

g *Low platelet counts (< 100 10^3^/mm^3^) likely artifactual due to platelet clumping*.

h *Low lymphocyte counts (< 1,000 /μl) likely attributable to stress responses*.

Archived serum from a subset of animals (33 spotted seals, 45 ribbon seals, and 5 bearded seals) was analyzed in 2017 for selected serum chemistry parameters (Table 2) at a commercial veterinary diagnostic laboratory (URIKA LLC, Bothell, Washington 98011) with a Beckman Coulter AU2700 Chemistry Analyzer (Beckman Coulter, Brea, California 92821). Marked to moderate hemolysis was noted in 3 of 83 samples (1 spotted seal, 2 ribbon seals). Because of the long interval between sample collection and analysis, only parameters known to be stable in samples stored at ultralow temperatures were included in the panel.

**Table 2:** Summary serum chemistry data for live-sampled spotted seals, ribbon seals, bearded seals, and ringed seals.

	**Spotted seal**			**Ribbon seal**		**Bearded seal**		**Ringed seal**	
**Source population**	**Rehabilitated: ALSC**	**Captive:LML**	**Wild caught:bering sea^a^ (MML)**	**Rehabilitated: ASLC**	**Wild caught:bering sea^a^(MML)**	**Wild caught: ASLC**	**Wild caughtChukchi sea, bering sea^a^ (MML)**	**Rehabilitated: ASLC**	**Captive:ASLC, LML**
Blood chemistry values	Total range	Total range	5–95% range	Total range	5–95% range	Total range	Total range	Total range	Total range
Number individuals	*n* = 9	*n* = 2	*n* = 33	*n* = 1	*n* = 45	*n* = 2	*n* = 5	*n* = 10	*n* = 3
Number samples	2–18	4–6	33	2–3	45	4	5	8–18	4–6
Individuals—age	<1.5 y	3–5y	Mixed^b^	1 adult	Mixed^c^	<1y	Mixed^d^	<1.5 y	mixed^e^
Individuals—sex	4 F, 5 M	2 M	20 F, 13 M	1F	24 F, 21 M	2 M	4 F, 1 M	3 F, 7 M	1 F, 2 M
Total protein (g/dl)	4.9–7.3	6.2–8.0	5.2–8.0	7.3–8.0	5.5–9.4	6.6–7.2	5.7–7.3	6.0–8.7	5.3–7.3
Albumin (g/dl)	1.7–3.5	3.0–3.9	3.2–4.1	3.0–4.4	3.3–5.0	3.0–3.3	3.2–4.0	2.6–4.0	2.0–4.0
Globulin (g/dl)	2.7–4.1	3.0–4.5	1.8–4.2	3.1–4.3	2.0–4.3	3.5–4.1	2.0–3.3	3.1–5.8	2.6–4.4
Glucose (mg/dl)	101–173	111–197	114–243	132–192	118–210	123–175	104–164	95–184	111–162
BUN (mg/dl)	23–56	29–71		41–55		12–25		19–43	36–76
Creatinine (mg/dl)	0.4–0.9	0.5–1.4	0.4–1.9	1.2–1.5	0.4–1.6	0.7–0.9	0.5–0.7	0.34–0.75	0.7–1.1
Bilirubin (mg/dl)	0.1–3.0	0.1–0.4	0.0–1.2	0.6–0.7	0.03–0.8	0.1–0.7	0.1–0.3	0.1–1.8	0.1–1.1
Cholesterol (mg/dl)	183–426	236–392	168–559	200–303	189–489	180–341	168–258	212–414	170–396
Alk phos (U/l)	113–628	77–181		32–38		105–378		75–841	71–147
ALT (U/l)	23–95	25–82		17–37		62–88		43–212	63–129
AST (U/l)	56–275	41–412		36–59		55–162		55–267	80–361
GGT (U/l)	14–37	1.0–30		11–13		5.0–7.0		6.0–139	5–55
CK (U/l)	86–991	61–2,565		357–1,320		225–820		67–1,626	178–19,967
Calcium (mg/dl)	9–12	8.2–9.6	8.8–12	9.5–10	9.3–13	8.7–9.7	9.1–11	8.6–11	8.1–9.7
Phosphorus (mg/dl)	5.1–9.6	4.3–7.8	5.7–11	4.7–6.6	6–12	5.1–8.0	8.1–11	4.7–8.8	2.7–10
Sodium (mEq/l)	150–161	152–169	145–155	156–157	147–166	152–157	147–153	148–169	154–165
Potassium (mEq/l)	3.9–5.9	5.1–5.7	3.8–5.4	4.2–4.8	3.5–4.9	3.8–4.0	3.9–5.1	3.9–5.2	3.6–6.2
Chloride (mEq/l)	107–116	108–118	97–109	112–114	95–110	107–111	98–109	104–130	109–125
Iron (mcg/dl)			141–752		146–844		214–793	
Magnesium (mg/dl)		1.5–2.1	1.8–2.8		1.9–2.7		2.0–2.8		1.8–2.4
Fibrinogen (mg/dl)	200–300							100–400	
Triglycerides (mg/dl)	22–54	121–336		21–56		34–85		22–117	54–259
Amylase (U/l)	218–398	306–459		22–35		0–35		0–49	4–46
Lipase (U/I)		19–54	25–65						6–25
Urea (mg/dl)					28–68		20–38		
LDH (U/l)	1,873–2,800			815–1,476		857–3,520		1,516–3,691	

*For each source population, the reported values (total range for data sets with n < 30 or 5–95% range for data sets with n > 30), numbers of individuals sampled (n), number of samples evaluated, ages of sampled individuals, and sexes of sampled individuals are provided. Source populations of sampled seals are from rehabilitation (Alaska SeaLife Center, ASLC), long-term captive (ASLC or Long Marine Lab, LML), or wild capture (ASLC or NOAA Marine Mammal Lab, MML). Chemistry parameters were measured on one of several analyzers (see notations in text); thus, these can be considered combined, descriptive data, or viewed quantitatively by subgroup. Data for individual animals within each subject group are provided in Supplementary Table 2*.

a *Chemistry values determined from archival (frozen) serum; enzymes excluded due to predicted degradation*.

b *Age distribution of spotted seals wild-caught in the Bering Sea: 10 adults, 3 subadults, 13 young-of-year, 7 pups*.

c *Age distribution of ribbon seals wild-caught in the Bering Sea: 17 adults, 15 subadults, 7 young-of-year, 6 pups*.

d *Age distribution of bearded seals wild-caught in the Chukchi Sea, Bering Sea: 4 subadults, 1 pup*.

e *Age distribution of ringed seals sampled in long-term captivity, Alaska SeaLife Center and Long Marine Lab: 1 subadult, 2 adults*.

### Stranded Ice Seals

#### Source of Animals and Handling

**Figure 1:**
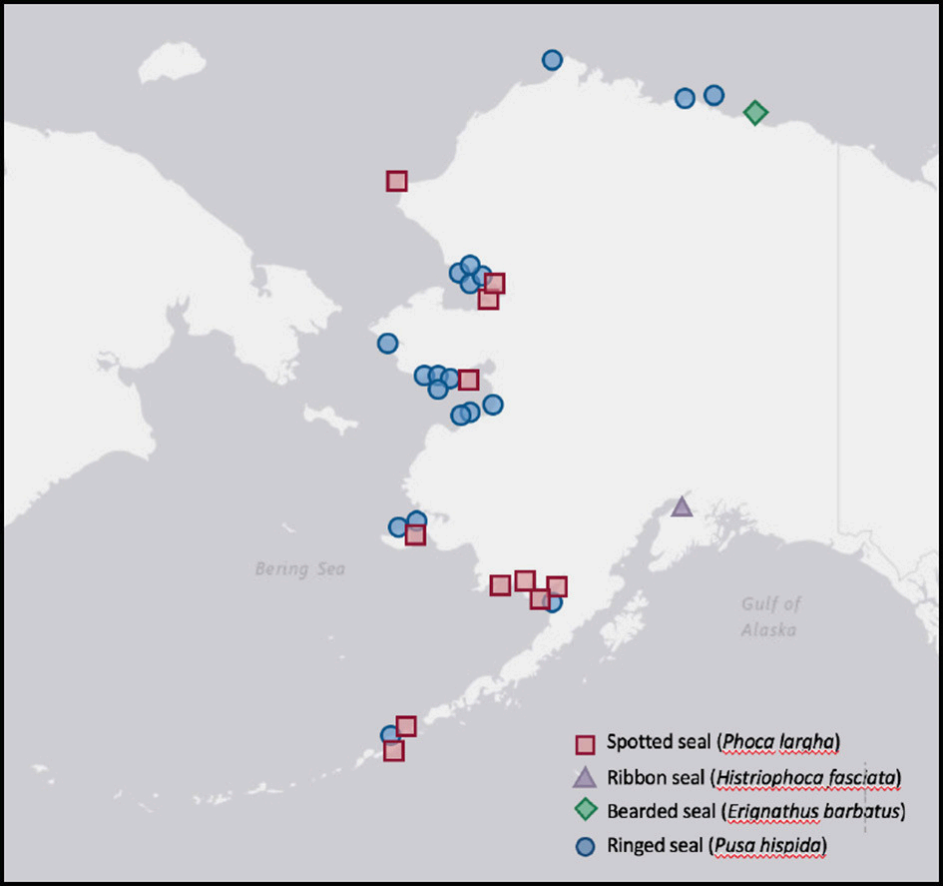
Stranding locations of Alaskan ice seals tested for serology (Table 4), microbial isolates (Table 5), and parasites (Table 6) at the Alaska SeaLife Center between September 2000 and March 2017. USGS National Map (https://viewer.nationalmap.gov).

Ice seals in distress (9 spotted, 1 ribbon, 1 bearded, 19 ringed; Table 3) from across Alaska (Figure 1) were reported to and assessed by staff from the Alaska SeaLife Center prior to being admitted for care in coordination with NOAA's Marine Mammal Health and Stranding Response Program. Cases from 2000 to 2017 are included here. As part of their medical care, seals received initial and periodical physical examinations under physical restraint or light sedation (butorphanol 0.16–0.70 mg/kg with midazolam 0.15–0.55 mg/kg IM) to obtain biological samples for routine diagnostic analysis and disease screening. If animals died or were euthanized because of the severity of their presenting problems, additional samples were obtained post-mortem. Morphological characteristics, including size, coat condition, presence of an umbilical remnant, and time of year, were used to distinguish between age groups.

**Table 3:** Case demographics of Alaskan ice seals tested for serology (Table 4), microbes (Table 5), and parasites (Table 6) at the Alaska SeaLife Center.

	**Spotted seal**	**Ribbon seal**	**Bearded seal**	**Ringed seal**
Live strand	9	1	1	19
Wild caught	0	0	2	0
Males	5	0	2	10
Females	4	1	1	9
< 1.5 Years old	9	0	3	19
Adult	0	1	0	0
Dead	0	0	1	8
Survivors	9	1	2	11
Total Individuals	9	1	3	19

*Individuals were live stranded with the exception of two bearded seals collected from the wild for long-term captive research. Other than one adult ribbon seal, individuals of all species were young-of-year and yearlings. Alaskan stranding locations corresponding to these cases are provided in Figure 1. Animals listed as Dead were deceased on arrival, died while under care, or were euthanized. Survivors are animals that were returned to health and released or retained in long-term captive care*.

#### Sample Collection, Processing, and Storage

Blood was collected from the lumbar region of the extradural intravertebral vein using 18–20-gauge, 1.5–3.5 inch needles, depending on the size of the seal, directly into plain, SST, and K_2_EDTA Vacutainer® tubes (BD Biosciences, Franklin Lakes, New Jersey, 07417, USA) or using a syringe and immediately transferring the blood into appropriate tubes. Whole blood was kept at room temperature and analyzed within 4 h. Plain and SST tubes were allowed to clot at room temperature then serum was separated by centrifugation for 5 min at 4,000 rpm. Serum was analyzed for chemistries or pipetted into 1 ml aliquots in cryovials and stored in a −80°C ultralow freezer until sent for further analysis. Swabs of conjunctiva and nasal passages were stored in viral transport media (Remel MicroTest M4RT Kit, Medex Supply, Passaic, New Jersey 07055) and frozen at −20°C until submitted for viral screening. Swabs of the rectum, wounds, abscesses, ears, or nares from live animals or swabs of internal organs of dead animals were collected and placed in Cary-Blair media (Becton, Dickinson & Company, Franklin Lakes, New Jersey, 07417, USA), refrigerated, and sent within 24 h to a commercial microbiology laboratory to screen for fecal pathogens or to isolate pathogenic bacteria from lesions. Fresh fecal samples were obtained opportunistically and refrigerated until on-site analysis within 4 h. While most samples for disease testing were obtained as part of admit sampling, some samples were collected later in the rehabilitation process as diseases became clinically evident or during necropsy. Tissues collected post-mortem were stored in neutral buffered formalin and later processed into slides for histopathological analysis.

#### Laboratory Analysis

CBC, serum chemistries, fecal parasite, and urine analyses were performed at the Alaska SeaLife Center. Only results from clinically healthy animals (9 spotted seals, 1 ribbon seal, 2 bearded seals, 10 ringed seals) were used to generate the hematology and serum chemistry tables in this report with no more than two sampling events per individual. Two microhematocrit tubes were centrifuged for 6 min at 14,000 rpm to determine Hct using a micro-capillary reader (±0.5%). Additionally, buffy coat smears obtained from spun microhematocrit tubes were examined microscopically to check for microfilaria (42). Blood smears were prepared, dried, fixed in methanol, and stained with Wright-Giemsa (Henry Schein Animal Health, Dublin, Ohio 43017) for manual white blood cell differential counts and to screen for hemoparasites. Complete blood counts were performed on an IDEXX ProCyte Dx, Heska CBC Diff, or QBC Hematology Analyzer using whole blood. Serum chemistries were performed using an IDEXX VetTest 8008.

Serum and molecular samples from the same individuals were shipped in batches about once a year on dry ice via express delivery screen for disease or exposure to disease agents. Reference laboratories and research facilities included the Oklahoma Animal Disease Diagnostic Laboratory, the Athens Veterinary Diagnostic Laboratory at the University of Georgia, University of California Davis School of Veterinary Medicine, the Animal Health Diagnostic Center at Cornell University, Kansas State Veterinary Diagnostic Laboratory, the Veterinary Diagnostic Laboratories at Colorado State University, and the Centers for Disease Control and Prevention. Thresholds established by the individual laboratories were used to determine evidence of prior natural exposure. A subset of seals were tested for microbial isolates and parasites. Culture swabs were submitted for aerobic bacterial culture at Providence Alaska Medical Center, Phoenix Central Laboratory, or the University of California Davis Veterinary Medical Teaching Hospital Microbiology Laboratory. Fecal pathogen screens for common enteric pathogens included *Salmonella, Shigella, Campylobacter jejuni*, and *Escherichia coli* O157. Identifications were performed on site with a Biomerieux Vitek2 GNI card or the Vitek2 NHI card (bioMérieux,Marcy-l′Etoile, France) if the organism was *Campylobacter*. Fecal samples were examined for ova and parasites using direct, float (Fecasol, Henry Schein, Melville, NY), and Baerman techniques. Other parasites were obtained by manual removal of external parasites from live animals and during necropsy. Some whole specimens or photomicrographs were sent to specialists for speciation (see acknowledgments). When available, fresh urine obtained from clean, dry surfaces was analyzed using the Urispec 11-Way Test Strips (Henry Schein Animal Health, Dublin, Ohio 43017) and a refractometer was used to determine urine specific gravity. Only urinalysis results from clinically healthy animals were used in this report. Tissue slides were examined by a pathologist (see acknowledgments) who provided histopathological diagnoses.

### Captive Ice Seals

#### Source of Animals and Handling

Three ringed seals and four spotted seals were stranded animals that had been rehabilitated at the Alaska SeaLife Center between 2010 and 2017 but were declared non-releasable by NOAA because rehabilitation took place outside of their home range. These animals were fully recovered with no substantive medical problems. Two bearded seals were obtained in 2015 and 2016 in Kotzebue, Alaska as part of a directed capture effort to support long-term research. Captive seals participated in established husbandry programs at the Alaska SeaLife Center in Seward, Alaska or Long Marine Laboratory, in Santa Cruz, California. As part of their medical care, seals received periodic physical examinations under physical restraint or light sedation (butorphanol 0.16–0.70 mg/kg with midazolam 0.15–0.55 mg/kg IM, or diazepam 0.26–0.42 mg/kg) to obtain biological specimens for routine diagnostic and disease screening analysis.

#### Sample Collection, Processing, and Storage

Biological samples obtained from long-term captive seals were collected, processed, and stored in the same manner as described above for stranded animals.

#### Laboratory Analysis

Biological samples obtained at the Alaska SeaLife Center were analyzed in the same manner as described above for stranded animals. Whole blood and serum obtained at Long Marine Laboratory were analyzed with a Bayer Advia 120 multispecies hematology system and an Olympus AU640e blood chemistry analyzer at Antech Diagnostics (Santa Clara, California 95051) within 24 h of collection.

### Analysis

Blood parameters are reported as either total ranges or trimmed ranges for each species and subject group. Where sample size is < 30 individuals, minimum to maximum values are used. Where sample size is >30 individuals, 5–95% data intervals are used to reduce the influence of outliers. Hematology and serum chemistry parameters are not reported as formal species-typical reference intervals for veterinary applications, although in some cases there are sufficient underlying data to generate such intervals (43).

Hematology and serum chemistry data were evaluated using Prism 7.0 (GraphPad Software, Irvine, California 92618) and compared qualitatively among the target species or to available data for harbor seals (38). Particular note was taken when the upper value of a given parameter was 20% higher or the entire range was higher than the values reported for harbor seals; almost no lower range values reported for Alaskan ice seals were below those reported for harbor seals. Because different laboratories and methods were used for logistical reasons, cautious indirect observational comparisons rather than statistical comparisons of blood analyte data were made within and among species.

Additional health assessment data from the subset of Alaskan seals handled during rehabilitation are reported for the target species as available and compared descriptively.

### Results

Summary data for hematology (Table 1) and serum chemistry parameters (Table 2) are presented for spotted, ribbon, bearded, and ringed seals as total (*n* < 30) or 5–95% (*n* > 30) ranges. The demographics for each subject group are included in the table headers. Corresponding metadata and blood panels for individual animals are provided in Supplementary Tables 1, 2, respectively.

Data concerning infectious disease exposure, microbiology, and parasites are reviewed for stranded ice seals from across Alaska (Figure 1) undergoing rehabilitation at the Alaska SeaLife Center (Table 3), and from the two bearded seals captured for directed research projects.

Results of tests for exposure to diseases of concern are summarized in Table 4. Most seals were seronegative for tested diseases. All seals were seronegative for avian influenza, phocine distemper virus, dolphin morbillivirus, porpoise morbillivirus, *Neospora, Toxoplasma, Sarcocystis, Coxiella*, and rabies. One spotted seal had a suspect positive for avian influenza on PCR testing of its fecal sample, although nasal and ocular swabs were negative. None of the spotted seals, the one ribbon seal, one of three bearded seals, and three of 18 ringed seals tested for antibodies to *Brucella* were positive. Five individuals (1 spotted, 1 ribbon, 3 ringed seals) were positive for herpesvirus. Of these, two (1 spotted, 1 ringed) were positive at admit with rising titers that decreased with subsequent testing, while the other three had very low herpes titers. All seals were seronegative for *Leptosirosis canicola, L. grippo*, and *L. pomona*. About a third of ringed seals (5 of 16) were positive for low levels of antibodies to *L. bratislava*. Some of these ringed seals were also positive for *L. hardjo* (3) and *L. ictero* (3), which was possibly due to a cross reaction. One spotted seal was positive for *L. hardjo*. All animals tested for antibodies to canine distemper virus and other morbillivirus at the Oklahoma Animal Disease Diagnostic Laboratory (OADDL) and the Athens Veterinary Diagnostic Laboratory (AVDL) (under the same PI, J. Saliki) were negative. One of the two bearded seals tested at Cornell's Animal Health Diagnostic Center was positive for antibodies to canine distemper virus but at a low dilution that decreased with subsequent testing; this may have been due to waning maternally acquired antibodies, an original false positive, or cross reaction—and therefore not necessarily due to disease.

**Table 4:** Disease exposure testing results for serology for Alaskan ice seals listed in Table 3.

**Infectious Agent**	**Spotted seal (*n* = 9)**	**Ribbon seal (n=1)**	**Bearded seal (*n* = 3)**	**Ringed seal (*n* = 19)**	**Laboratory,^a^ method,^b^ (References)**
**MISCELLANEOUS**					
Avian influenza	0/8 (0%)	–	0/3 (0%)	0/11 (0%)	Runstadler, ELISA (44, 45)
	1/5 (20%)	–	0/2 (0%)	0/3 (0%)	Runstadler, rtPCR (45)
*Brucella*	0/8 (0%)	1/1 (100%)	–	1/9 (11%)	Mystic, cELISA (46)
	–	–	1/3 (33%)	2/9 (22%)	OADDL, Card (47)
*Coxiella*	0/6 (0%)	0/1 (0%) CF	0/3 (0%)	0/15 (0%)	CDC or CSU, IFAT (48)
Herpesvirus PhHV-1	1/6 (17%)	1/1 (100%)	0/1 (0%)	2/12 (17%)	OADDL or AVDL, SN (49)
	0/2 (0%)	–	0/2 (0%)	1/3 (33%)	UC-Davis, ELISA (50)
Rabies	–	–	–	0/14 (0%)	KSU, RFFIT (56)
***LEPTOSPIRA***					
*L. bratislava*	0/9 (0%)	0/1 (0%)	0/3 (0%)	5/16 (31%)	OADDL or AVDL, MAT (51)
*L. canicola*	0/9 (0%)	0/1 (0%)	0/3 (0%)	0/16 (0%)	OADDL or AVDL, MAT (51)
*L. grippo*	0/9 (0%)	0/1 (0%)	0/3 (0%)	0/16 (0%)	OADDL or AVDL, MAT (51)
*L. hardjo*	1/9 (11%)	0/1 (0%)	0/3 (0%)	3/16 (19%)	OADDL or AVDL, MAT (51)
*L. ictero*	0/9 (0%)	0/1 (0%)	0/3 (0%)	3/16 (19%)	OADDL or AVDL, MAT (51)
*L. pomona*	0/9 (0%)	0/1 (0%)	0/3 (0%)	0/16 (0%)	OADDL or AVDL, MAT (51)
**MORBILLIVIRUS**					
Canine distemper virus	0/9 (0%)	0/1 (0%)	0/1 (0%)	0/16 (0%)	OADDL or AVDL, VNT (52)
	–	–	1/2 (50%)	–	Cornell, SN (53)
Phocine distemper virus	0/9 (0%)	0/1 (0%)	0/3 (0%)	0/16 (0%)	OADDL or AVDL, VNT (52)
Dolphin morbillivirus	0/2 (0%)	–	0/1 (0%)	0/10 (0%)	OADDL or AVDL, VNT (52)
Porpoise morbillivirus	0/1 (0%)	–	0/1 (0%)	0/10 (0%)	OADDL or AVDL, VNT (52)
**PROTOZOA**					
*Neospora*	0/5 (0%)	–	0/2 (0%)	0/3 (0%)	UCDavis, IFAT (54)
*Toxoplasma gondii*	–	–	0/1 (0%)	0/9 (0%)	OADDL, Latex agglutination (55)
	0/5 (0%)		0/2 IFA (0%)	0/3 (0%)	UCDavis, IFAT (54)
*Sarcocystis*	0/5 (0%)	–	0/2 (0%)	0/3 (0%)	UCDavis, IFAT (54)

*Results are provided as number of individuals that tested positive out of number of individuals tested, and % prevalence*.

a *Laboratory abbreviations*:

*Runstadler, Runstadler Laboratory, Tufts University, 200 Westboro Rd, North Grafton, MA 01536 USA*.

*Mystic, Mystic Aquarium, 55 Coogan Blvd, Mystic, CT 06355 USA*.

*OADDL, Oklahoma Animal Disease Diagnostic Laboratory, W Farm Rd, Stillwater, OK 74078 USA*.

*AVDL, Athens Veterinary Diagnostic Laboratory, University of Georgia, 501 DW Brooks Dr., Athens, GA 30602 USA*.

*UCDavis, University of California Davis School of Veterinary Medicine, One Shields Avenue, Davis, CA 95616 USA*.

*Cornell, Animal Health Diagnostic Center, Cornell University, Ithaca, NY 14853-6401 USA*.

*KSU, Kansas State Veterinary Diagnostic Laboratory 1800 Denison Ave, Manhattan, KS 66506 USA*.

*CDC, Centers for Disease Control and Prevention, 1600 Clifton Road Atlanta, GA 30329-4027 USA*.

*CSU, Veterinary Diagnostic Laboratories, Colorado State University, 300 West Drake Road, Fort Collins, CO 80526 USA*.

b *Method abbreviations*:

*ELISA, enzyme-linked immunosorbent assay*.

*IFAT, immunofluorescence antibody test*.

*MAT, microscopic agglutination test*.

*SN, serum neutralization test*.

*RFFIT, rapid fluorescent focus inhibition test*.

*rtPCR, real time polymerase chain reaction*.

*VNT, virus neutralization test*.

Results from microbial cultures are summarized in Table 5. A minority of animals (5 of 18) screened for pathogens from fecal samples, across all species, were positive. Seals positive for fecal pathogens were also positive on additional microbial testing. Most additional microbial isolates listed for spotted seals came from a single individual with only one other animal growing an isolate of concern; both of these spotted seals were very ill but survived rehabilitation. In 10 ringed seals, isolates of concern were detected in wounds, abscesses, ears, or nares or from swabs of internal organs obtained post-mortem. One additional ringed seal was diagnosed with bacterial pneumonia based on radiographs and clinical symptoms, however no specific isolate was identified. In total, 11 of 19 ringed seals were impacted by bacterial infection.

**Table 5:** Microbial isolates identified in Alaskan ice seals listed in Table 3.

	**Spotted seal (*n* = 7)**	**Ribbon seal (*n* = 1)**	**Bearded seal (*n* = 1)**	**Ringed seal (*n* = 14)**
Positive for fecal pathogens isolates listed below	1/7 (14%)	0/1 (0%)	0/1 (0%)	4/9 (44%)
Positive for isolates from other locations, isolates listed	2/7 (29%)	0/1 (0%)	1/1 (100%)	10/14 (71%)
below				
No microbial cultures done, diagnosed with bacterial	–	–	–	1
pneumonia				
No microbial cultures done, no disease concerns	2	–	2	5
**ISOLATES**				
*Aeromonas* sp.	Feces, wound	–	–	–
*Aeromonas hydrophila*	–	–	–	Ear, wound^*^, abscess
*Aeromonas veronii*	–	–	–	Feces
*Candida parapsilosis*	–	–	–	Skin^*^
*Citrobacter freundii*	–	–	–	Ascites^*^
*Clostridium sp*	–	–	–	Ascites^*^
*Corynebacterium sp*.	–	–	–	Skin
*Escherichia coli*	–	–	–	Ascites^*^, ear
*Edwardsiella tarda*	Wound	–	–	–
*Enterococcus faecium*	Blood	–	–	–
*Enterococcus sp*.	–	–	–	Ascites^*^, lung^*^, wound
*Enterobacter cloacae*	Wound	–	–	Nares, kidney^*^, ear, ascites^*^
*Fusobacterium mortiferum*	–	–	–	Abscess
*Hafna alvei*	–	–	–	Abscess
*Klebsiella pneumoniae*	–	–	–	Feces, lung^*^
*Madurella grisa*	–	–	–	Hair
*Methylobacterium sp*	Blood	–	–	–
*Plesiomonas shigelloides*	Feces	–	–	Lung^*^, feces
*Pseudomonas aeruginosa*	Abscess	–	Lung^*^, CSF^*^, hilar^*^ lymph node^*^, pericardium^*^	Lung^*^, ear
*Pseudomonas sp*.	Wound	–	–	–
*Psychrobacter phenylpyruvicus*	–	–	–	Skin^*^
*Prevotella oralis*	–	–	–	Abscess
*Riemerella anatipestifer*	–	–	–	Skin
*Shewanella algae*	–	–	–	Feces
*Staphylococcus sp*.	Abscess	–	–	–
*Staphylococcus sp*. (coagulase +)	–	–	–	Nares, wound
*Staphylococcus aureus*	Wound	–	–	–
*Streptococcus phocae*	–	–	–	Blood, abscess, LN^*^
*Streptococcus sp*. (β-hemolytic)	Abscess, wound	–	–	Wound
*Vibrio alginolyticus*	–	–	–	Ascites^*^
*Vibrio cholerae*	Feces	–	–	–

*Results for fecal and other pathogens are provided as number of individuals that tested positive out of number of individuals tested, and % prevalence*.

* *Indicates isolates from swabs obtained at necropsy*.

Results from parasitic examinations are summarized in Table 6. Half the spotted seals (5 of 9) were negative for ova and parasites on fecal exams done by any method. In contrast, parasites were found in most ringed seals (12 of 16) through fecal (any method) or other exams (grossly visible, revealed through blood tests, or microscopic review of tissues). Gastrointestinal nematodes were seen in all species. Seal lice (*Echinophthirius horridus)* and microfilaria (one was identified as *Acanthocheilonema spirocauda;* the rest were not speciated) were only seen in ringed seals.

**Table 6:** Parasites identified in Alaskan ice seals listed in Table 3.

	**Spotted seal (*n* = 9)**	**Ribbon seal (*n* = 1)**	**Bearded seal (*n* = 3)**	**Ringed seal (*n* = 16)**
Negative fecal exams for ova & parasites and/or	5/9	0	0	4/16
none seen on post-mortem or other exam				
Positive fecal exams, parasite listed below	4/9	1	3	5/16
Parasite noted on post-mortem or other exam	–	–	–	9/16
No exam for parasites performed	–	–	–	3
**TREMATODES (FLUKES, NOT SPECIATED)**				
Any trematode	1/9 (feces)	0/1	0/3	0/0
**CESTODES (TAPEWORMS)**				
*Anophryocephalus sp*	1/9 (feces)			
*Diphyllobothrium sp*	1/9 (feces)		1/3 (feces)	
Cestode, not speciated			2/3 (feces, GI^*^)	4/16 (feces, GI^*^)
Any cestode	2/9	0/1	2/3	4/16
**NEMATODES (ROUNDWORMS)**				
**Filarioids**				
*Acanthocheilonema spirocauda*				1/16 (blood)
Microfilaria, not speciated				3/16 (blood)
**Strongylids (hookworms)**				
*Otostrongylus circumlitus*			1/3 (feces)	2/16 (lung^*^)
Larva in feces, presumably lungworm	1/9 (feces)		1/3 (feces)	3/16 (feces)
**Ascarids**				
*Anisakis sp*.	1/9 (feces)			1/16 (GI)
*Contracaecum sp*.	1/9 (feces)			2/16 (feces, GI^*^)
*Pseudoterranova decipiens*	1/9 (feces)		1/3 (feces)
Not speciated			1/3 (feces)	1/16 (GI)
Larva, not speciated	1/9 (feces)			1/16 (lymph node^*^)
Nematode, not speciated	1/9 (feces)	1/1 (feces)	2/3 (feces, GI^*^)	3/16 (feces, GI^*^)
Any nematode	4/9	1/1	3/3	11/16
**ACANTHOCEPHALA (THORNY-HEADED WORMS)**				
*Corynosoma strumosum*				1/16 (GI^*^)
*Corynosoma semerme*				1/16 (GI^*^)
Acanthocephala, not speciated	1/9 (feces)			2/16 (feces, GI^*^)
Any acanthocephala	1/9	0/1	0/3	3/16
**EXTERNAL PARASITES**				
**Lice**				
*Echinophthirius horridus*				2/16 (skin)
**Mites**	1/9 (feces)			
**SUMMARY FINDINGS**				
Any parasites	4/9	1/1	3/3	12/16
Multiple parasites	4/9	0/1	3/3	9/16

*Location of parasites found included the gastrointestinal system (GI), feces, lymph node, lungs, blood, or skin during diagnostic tests for parasites or opportunistically found during other exams*.

* *Specimens obtained at necropsy*.

Ranges for urinary specific gravity for the different ice seal species are provided in Table 7 in comparison to the range seen in harbor seals (*Phoca vitulina*) treated at the same facility (C. Goertz, unpublished data).

**Table 7:** Specific gravity of urine obtained from Alaskan ice seals listed in Table 3, shown with comparative data from harbor seals evaluated at Alaska SeaLife Center.

	**Spotted seal**	**Ribbon seal**	**Bearded seal**	**Ringed seal**	**Harbor seal**
Number Animals	6	1	2	10	29
Number Samples	9	1	4	14	37
Minimum	1.018	1.035	1.040	1.010	1.004
Maximum	1.056	1.035	1.061	1.060	1.051
Average	1.041	1.035	1.046	1.036	1.025

Gross necropsy and histopathological examination of tissues from deceased ringed seals handled during rehabilitation confirmed pre-mortem diagnoses including both bacterial infections and parasitic infestations. Evidence of hepatic damage was found in some ringed seals which was consistent with derangements seen in serum enzymes associated with hepatobiliary function found during admit exams. Cases of dermal pathology were noted in ringed seals that died and those that survived, though none were afflicted with the debilitating clinical signs seen in other UME seals. Additional findings included sepsis, fat atrophy, and shock. Necropsies of spotted or ribbon seals did not occur as part of study activities.

One long-term captive bearded seal at Long Marine Laboratory died following traumatic injury. Histopathology for this nearly 2-year-old, apparently healthy individual showed systemic infection of multiple muscles (skeletal muscles, esophagus, tongue, and palate) with two forms of apicomplexan protozoal tissue cysts, in association with myositis and myophagia. A single cyst was observed in the spinal cord, with no associated inflammation. The mature cysts, mainly within myofibers, were morphologically compatible with *Sarcocystis spp*. and suggestive of subacute to chronic infection. The seal also exhibited patchy to diffuse, superficial dermal mycosis attributed to dermatophytosis and/or dermal candidiasis. Attempts at fungal isolation from cryopreserved skin were unsuccessful. As the seal died during the annual molt, it is unknown whether this condition may have been related to observed perimortem hair loss. Affected areas of skin were characterized by patchy invasion of the stratum corneum by fine, branching septate (and possibly, aseptate) fungal structures suggestive of fungal hyphae and pseudohyphae.

### Discussion

Knowledge of typical health indicators is an invaluable tool to assess the health of individuals and populations. The information presented here provides a set of initial baseline parameters and observations for Alaskan ice seals that may be considered in the context of emerging conservation concerns for Arctic environments, including those related to climate change and associated sea ice loss, increasing risks of contamination from oil, hazardous materials, and pollutants, introduction of pathogenic vectors including parasites and viruses, and food safety issues for subsistence communities. A discussion of key findings from the free-ranging, short-term rehabilitation, and long-term captive individuals sampled for this study follows.

Red blood cell (RBC) parameters, particularly hemoglobin (Hb) and hematocrit (Hct), are primary indicators of oxygen storage and transport capacity in marine mammals, which have substantially larger oxygen stores than their terrestrial counterparts (57). In particular, phocid seals have impressive blood oxygen stores to support prolonged breath-hold diving, with the longest and deepest diving species generally having the largest stores (58). The four species we evaluated exhibited higher values for all RBC parameters (RBC, Hb, Hct, MCV, MCH, MCHC) than more temperate living harbor seals (59, 60). When compared to other Arctic seals, the Alaskan ice seals exhibited higher RBC counts than both harp and hooded seals, and higher Hct values than harp seals (61, 62). Most notably, ribbon seals had the highest Hct values of all Arctic seals, reaching ~75% in both captive and wild individuals. Although data on free-ranging diving behavior are limited for many Arctic phocids, high Hct, and Hb indicate that ribbon seals, the presumed deepest diver of the sampled species, also have the highest blood oxygen storage capacities. Among all Arctic seals, hooded seals are known to make the longest and deepest foraging dives, yet previously published Hb and Hct data for this species (61, 62) are lower than our values for ribbon seals. It is possible that the larger body size and robust muscle oxygen stores of hooded seals further contribute to their impressive dive profiles (63). Although there are clear differences in species-typical red blood cell indices for the target species, which vary widely in body size and routine foraging behavior, all appear to have robust blood oxygen storage capacities. This is consistent with the thick, dark, sludgy appearance of whole blood obtained from these seals as compared to other pinnipeds.

Given that RBC indices in these species are higher than in most pinnipeds, certain conditions such as anemia may go unrecognized. Additionally, the higher RBC counts along with higher hemoglobin and iron may impact other physiological processes and serum chemistry parameters even in health (64) which may complicate veterinary assessments (i.e., hydration status, kidney function). Greater attention to RBC morphology, consistency of RBC size and color, presence of nucleated RBCs, plasma color, and reticulocyte count (not reported here) may be important, in addition to having robust age and sex specific data sets.

With respect to typical white blood cell (WBC) counts, Alaskan ice seals had more leukocytes than other seal species (60). Alaskan ice seals exhibited similar proportions of WBC types as other pinnipeds, with more neutrophils than lymphocytes and far fewer monocytes and eosinophils. Some individuals had low lymphocyte counts (<1,000/μl) which may be attributable to stress. Ringed seals had the highest eosinophil counts, which may correspond to a possible higher rate of parasitism in this species (see later).

Serum chemistry parameters aid in the evaluation of disease, contaminant exposure, and abnormal physiological conditions but can also change with normal development, feeding, geography, and reproductive state. In general, ice seals had similar ranges as other pinnipeds (60) though several had higher upper values. Ribbon seals had the highest total protein and albumin of the species sampled. Rehabilitated ringed seals had higher globulins; this is likely related to heightened immune stimulation and/or antigen exposure at admit from underlying disease that that was not observed in the other species.

Elevations of some blood chemistry parameters in the rehabilitation groups can be attributed to normal development of primarily young individuals (65), namely bilirubin (neonatal fetal hemoglobin turnover) and alkaline phosphatase, calcium, and phosphorus (upregulated secondary to bone growth). Several hepatobiliary-associated parameters (bilirubin, ALT, AST, and GGT) overlapped but had notably higher upper ranges in the stranded seals sampled as compared to wild Pacific harbor seal pups (39). These elevations in rehabilitated ringed seals are probably reflective of prolonged recovery from hepatobiliary disorders noted at admit and secondary to their profound morbidity and aberrant parasite migration. Alternatively, or additionally, these parameters and creatine kinase (CK) may relate to how animals are captured or handled for blood draws.

Differences in glucose, blood urea nitrogen (BUN), cholesterol, calcium, and potassium compared to other pinnipeds were considered negligible or not of physiological significance. Higher levels of sodium, and to a lesser extent chloride, were seen in spotted, ribbon, and ringed seals compared to other pinnipeds. The higher urinary specific gravity seen in Alaskan ice seals is consistent with these seals tending to have higher electrolytes, in particular sodium. As sodium levels increase, kidneys are stimulated to retain water resulting in more concentrated urine (66).

Consistent with other studies (32–35, 67–72), serological testing of Alaskan ice seals handled in rehabilitation or shortly after capture revealed little to no evidence of exposure to most diseases. While the young of the year animals in this report would not have had sufficient time in the wild to produce their own antibodies, they would have carried maternally derived antibodies. In the present study, all animals were seronegative for avian influenza, dolphin morbillivirus, phocine distemper virus, porpoise morbillivirus, *Neospora, Toxoplasma, Sarcocystis, Coxiella*, and rabies. It is notable that evidence of exposure to phocine distemper virus was not detected, although antibodies to the virus have been found in other marine mammals in some areas of the North Pacific [for review see (73)]. The few very low level positive results for antibodies to three *Leptospira* (*L. bratislava, L. hardjo*, and *L. ictero*), as well as the one positive result for antibodies to canine distemper, were not high enough to be consistent with disease, nor were they associated with clinical signs of infection. These findings suggest these diseases are not currently present in Alaskan ice seals, and also may indicate that these populations are immunologically naive and therefore potentially susceptible to them if introduced. An exception appears to be phocine herpesvirus and *Brucella*. The level of seroprevalence to *Brucella* is in accordance with other regional studies (32–35, 74), and while antibodies to *Brucella* were present, no seals had clinical disease. We found low to moderate levels of antibodies to phocine herpes PhHV-1 in a few stranded spotted, ribbon, and ringed seals. Two seals had rising titers on successive samples, suggesting recent disease at the time of admission, but none had clinical signs associated with herpesvirus. Chronic exposure to phocine herpes strains PhHV-1 and PhHV-2 has now been reported for spotted, ribbon, bearded, and ringed seals in the North Pacific, indicating that marine mammals in this region may be regularly exposed to the virus (32, 34, 35, 75). Prevalence and/or relative titers of PhHV-1 could be a useful adjunct to evaluating individual and population health status, as herpes has been shown to manifest more strongly in animal populations with limited resources or compromised health status (76, 77) as well as different levels of mental stress in people (78).

Bacterial isolates were more commonly obtained from ringed seals than spotted seals admitted for rehabilitation, which is consistent with greater morbidity observed in the ringed seals. More than half the ringed seal cases had clinically significant microbial infection; of these, most ultimately died or were euthanized because of the severity of their presenting problems. Isolates from spotted seals came from just two individuals that were profoundly ill prior to fully recovering from their presenting problems. In fact, all nine spotted seals handled for rehabilitation fully recovered. The single bearded seal admitted for rehabilitation succumbed to sepsis, with *Pseudomonas aeruginosa* isolated from multiple tissues. No bacterial isolates of concern were identified in the ribbon seal that was found out of range or in the two healthy bearded seals that were collected for research. Few antibiotic sensitivity tests were performed, however those results showed high sensitivity to all antibiotics tested.

There are several datasets concerning parasites in Alaskan ice seals, summarized by Geraci and St Aubin (79), Boveng et al. (13, 14), Cameron et al. (19), and Kelly et al. (18). In addition, as part of a survey for parasites in subsistence-harvested Alaskan seals (2 spotted, 36 bearded, 2 ribbon, 13 ringed seals), Tuomi et al. (personal communication, P. Tuomi) examined multiple samples (feces, gall bladder, heart, intestines, liver, lung, spleen, and stomach) for ova, larva, and whole parasites [see also (80)]. No parasites were found in the hearts or spleens of any individual sampled. Parasites were infrequently found in lungs; two bearded seals had anisakid nematodes (likely originating from the gastro-intestinal system), one ringed seal had a metastrongylid, and one spotted seal had a filaroid. While few spotted and ribbon seals were studied, all were found to have anisakids or acanthocephalalids in the stomach or intestines. Bearded seals had the greatest percentage of parasites with 81% of stomachs containing anisakids and 36% containing cestodes; 53% of gall bladder samples contained trematodes, and intestinal samples contained cestodes (83%), anisakid (11%), trematodes (6%), and acanthocephalans (3%). Bearded seal fecal samples had a similar complement of parasites with the addition of metastrongylids (6%) but no trematodes. In contrast to the present study, the ringed seals evaluated by Tuomi had lower incidences of parasite infestations, with anisakids (8%), trematodes (8%), cestodes (15%), and protozoa (8%) found only in different portions of the gastrointestinal tract; the protozoan was a coccidia oocyst morphologically consistent with *Eimeria sp*.

Of the seals handled during rehabilitation or following capture in the present study, bearded seals and ringed seals were more heavily parasitized than spotted seals. Additionally, more ringed seals had clinically significant parasites. Microfilaria—an early life stage of some parasitic nematodes which are released into the blood by adult worms, taken up by blood-feeding arthropod vectors, and develop into infective larvae that are transmitted to a new host—were only seen in ringed seals. Lungworm infections were confirmed or presumed in five ringed seal cases (31%) and one spotted seal case (11%) as well as the one bearded seal stranding case. Seal lice, *E. horridus* can serve as an intermediate host of the seal heartworm, *A. spirocauda*, which was not confirmed in any of the animals examined as part of this study, but could have been the source of the microfilaria seen in blood in a few cases. The apicomplexan protozoa seen in the captive bearded seal that died may have been acquired after leaving Alaska; however, *Sarcocystis spp*. has been previously reported in this species in the North Pacific (81).

Various degrees of alopecia and other skin conditions were observed in several animals included in this report. None, however, were consistent with the alopecia seen in the more extreme UME cases which included ulcerations as well as more profound derangements of the skin. Instead, it is the consensus of veterinarians involved with rehabilitating these animals that the alopecia was secondary to other stressors such as external wounds, chronic pulmonary disease, heavy parasitism, and/or liver disease. Additional contributors to observed hair loss may include exposure to novel environments such as warmer year-round temperatures, substrates rougher than snow or ice, and light exposure cycles different from those found in natural habitats, even when seals are housed in natural sea water with no added disinfectants. Dermal mycosis as found in the captive bearded seal that died has not been reported in bearded seals, but has been associated with persistent fresh water exposure, relatively warm water temperatures, and stress associated with seasonal molt in other pinnipeds (82, 83). When compared to other pinnipeds housed at the same facilities, Arctic ice seals appear more prone to hair loss and irregular molt (C. Goertz, unpublished data, D. Casper, personal communication, T. Schmitt personal communication).

### Conclusions

The recent and unresolved unusual mortality event declared for Alaskan ice seals (2011–2018) is an alarming reminder of the challenges associated with assessing the health status of spotted, ribbon, bearded, and ringed seal populations. In particular, this event highlighted the sparsity of available data concerning health parameters and abundance estimates for these species. Given apparent low historical exposure and therefore low resistance to disease, recent extra-limital sightings of ice seals^1^, and projected increases in contact between individuals and populations caused by reduced availability of sea ice (e.g., 10), it is likely that risks of epizootic events will continue to increase for these species. While still limited with respect to sample sizes and demographic groups in some cases, the hematology, and serum chemistry data provide markers for normal health status and physiology. Similar studies for other vulnerable seal species have shown that such datasets can be consistent as more individuals are added (84). Along with case-specific findings concerning disease exposure, parasite loads, and microbiology, these observations of Alaskan ice seals contribute to a much needed expanded understanding of these species.

### Ethics Statement

This study was carried out in accordance with the requirements of federal marine mammal research permits 14535, 15142, 15126, 18902, 19309, or the Alaska SeaLife Center-NOAA/NMFS Stranding Agreement. Animal protocols for captive seals were approved by the Institutional Animal Care and Use Committee at the University of California Santa Cruz. Animal protocols for wild seals were approved under IACUC protocols A/NW 2010, A/NW 2016.

## Author Contributions

CG and CR devised the project. CG, CR, HZ, and PB were responsible for sample collection. CG, CR, NT, HZ, and PB contributed to evaluation of the data and manuscript preparation. CR and PB were responsible for funding, animal welfare considerations, and project oversight.

### Conflict of Interest Statement

The authors declare that the research was conducted in the absence of any commercial or financial relationships that could be construed as a potential conflict of interest.

## Footnotes

^1^For example, unusual sightings of ribbon seals in Wasilla and Yakutat (Alaska), and in Washington, Oregon, and California; increases in sightings and strandings of ringed seals around Unalaska Island (Aleutian Archipelago) and one stranding in Washington (National Marine Mammal Stranding Network, D. Fauquier, personal communication).
